# Cryptogenic HACEK (Haemophilus, Aggregatibacter, Cardiobacterium, Eikenella, and Kingella) CNS Abscess in an Otherwise Healthy Patient With a Patent Foramen Ovale

**DOI:** 10.7759/cureus.68471

**Published:** 2024-09-02

**Authors:** Ai Ohno, Matthew M Cappiello, Lu Pu, Daniel Rogstad

**Affiliations:** 1 Neurology, Loma Linda University Medical Center, Loma Linda, USA; 2 Infectious Diseases, Loma Linda University Medical Center, Loma Linda, USA

**Keywords:** cns actinomycosis, cryptogenic brain abscess, patent foramen oval, hacek group, actinomyces infection

## Abstract

*Actinomyces *and HACEK (*Haemophilus, Aggregatibacter, Cardiobacterium, Eikenella, *and *Kingella*) organisms are part of the oral microbiome and rarely affect the central nervous system (CNS). CNS infections with these agents can be caused by contiguous or hematogenous spread. We present a case of a 25-year-old immunocompetent male who presented with a one-week history of progressively worsening generalized headaches, photophobia, nausea, and vomiting. Despite a normal physical exam and the absence of leukocytosis, head imaging showed a right frontal lobe abscess. The patient was started empirically on vancomycin, ceftriaxone, and metronidazole, and surgery was performed. Surgical cultures grew organisms from the HACEK spectrum (*Aggregatibacter*, *Eikenella*),* Gemella morbillorum*, *Streptococcus constellatus*, and *Actinomyces georgiae*. Serial imaging studies showed a rapid increase in the size of the residual abscess, and the patient needed repeat intervention within five days. He was discharged five days after the repeat surgery on IV therapy prior to the transition to oral antibiotics. While the patient was found to have a small patent foramen ovale, there was no evidence of bacteremia or valvular vegetation, and no evidence of dental or sinus disease was seen on imaging. This case suggests that even in the absence of any clear sources of infection, cryptogenic brain abscesses can still occur sporadically in young, healthy patients.

## Introduction

Actinomycosis and HACEK (*Haemophilus, Aggregatibacter, Cardiobacterium, Eikenella, *and* Kingella*) group infections are classified as endogenous infections. The microbiologic agents causing these infections are commonly found on oropharyngeal mucosal surfaces and gain access to deeper tissue through disruption of the mucosal barrier by trauma, procedures, or foreign objects [1]. They rarely affect the central nervous system (CNS), infections of which are primarily caused by contiguous or hematogenous spread [2]. While the exact pathophysiology for CNS abscesses is complex, a four-stage histological model has been proposed, including early and late cerebritis and capsule formation [3]. Guidelines on therapy for CNS actinomycosis are lacking. The current consensus is surgical debridement followed by a prolonged course of intravenous antibiotics, followed by oral therapy for 6-12 months [2,4].

This article was previously presented as a poster at the 2024 American Academy of Neurology Annual Meeting on April 16, 2024.

## Case presentation

A 25-year-old immunocompetent male with no known past medical history presented to the emergency department with one week of progressively worsening generalized headaches, photophobia, nausea, and vomiting. Vital signs were stable without leukocytosis or focal neurological deficits on examination. He had good dentition and had no recent dental procedures. He did have a remote history of blunt head trauma in the setting of an assault one year prior to presentation, with no residual symptoms or known sequelae. He did not seek medical attention at that time.

**Figure 1:**
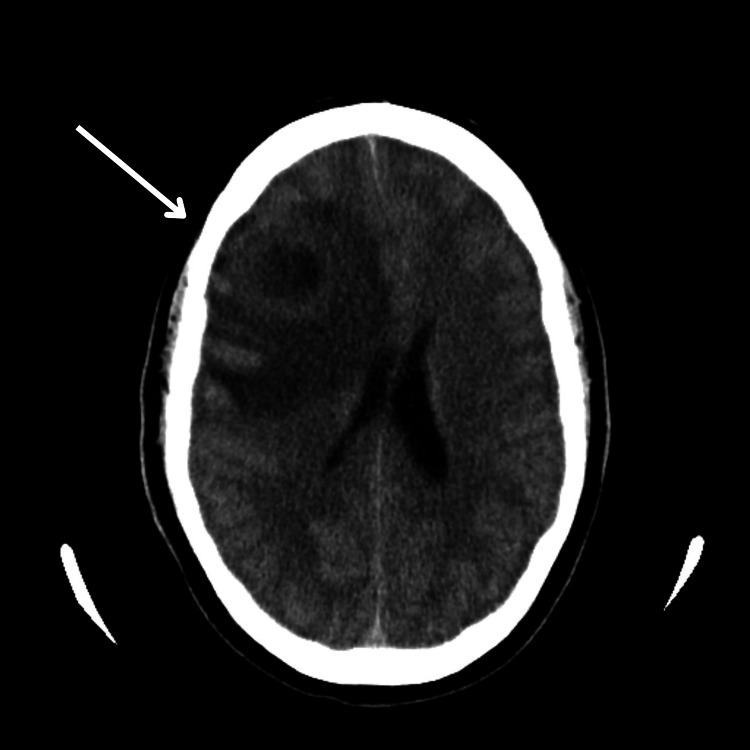
Preoperative head imaging CT head showing a 3.8 x 1.6 cm right frontal lobe abscess with a 7 mm leftward midline shift.

**Figure 2:**
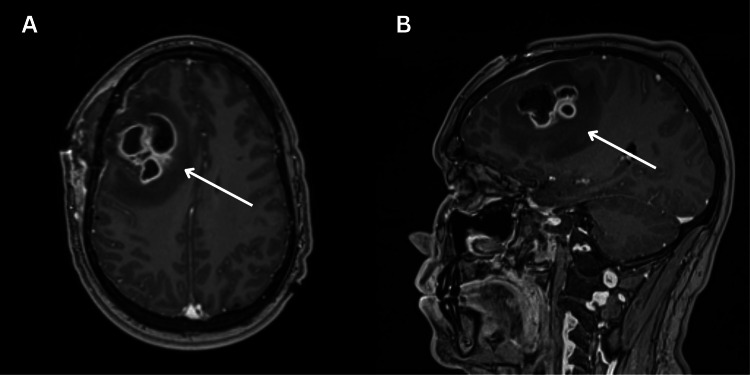
Postoperative head imaging Axial (A) and sagittal (B) view of a post-gadolinium T1-weighted brain MRI on postoperative day 4 showing a multi-loculated, rim-enhancing lesion (3.8 x 1.6 cm) in the right frontal lobe with regional mass effect and 6-7 mm leftward midline shift.

CT imaging revealed a 3.8 x 1.6 cm right frontal lobe abscess with a 7 mm midline shift (Figure 1). The patient was started empirically on vancomycin, ceftriaxone, and metronidazole. Evacuation and washout were performed. Small satellite abscesses were noted posterior to the main abscess, and a 1.3 cm residual abscess along the posterior aspect of the surgical cavity was noted on postoperative imaging. Repeat imaging studies showed a rapid increase in the size of the residual abscess to 3.8 cm on postoperative day (POD) 4 (Figure 2). The patient underwent repeat surgical intervention on POD 5, with findings of a thickened posterior capsular wall that was evacuated for a second time.

Imaging after the repeat surgery showed no residual abscess. Surgical cultures grew organisms from the HACEK group (*Aggregatibacter*, *Eikenella*), *Gemella morbillorum*, *Streptococcus constellatus*, and *Actinomyces georgiae*. While the patient was found to have a small patent foramen ovale (PFO), there was no evidence of bacteremia or valvular vegetation on the transesophageal echocardiogram. The patient did have an incidental, benign-appearing, non-infectious tongue lesion on the anterolateral surface, but no evidence of odontogenic abscess or sinus disease was seen on imaging.

**Figure 3:**
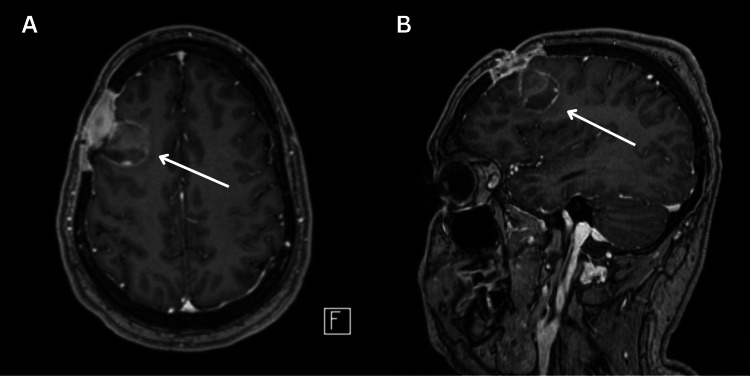
Head imaging at the three-month follow-up Axial (A) and sagittal (B) view of a post-gadolinium T1-weighted brain MRI at the three-month follow-up showing a small (3.0 x 3.4 cm) rim-enhancing collection within the right frontal lobe with internal diffusion restriction, which is likely related to hemorrhagic products.

**Figure 4:**
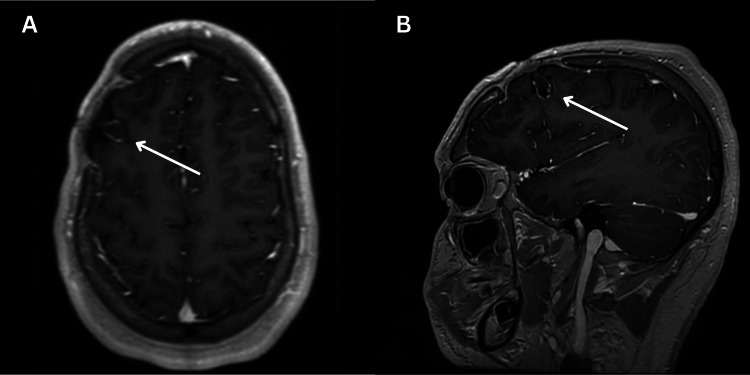
Head imaging at the eight-month follow-up Axial (A) and sagittal (B) view of a post-gadolinium T1-weighted brain MRI at the eight-month follow-up showing a small (9.3 x 8.6 mm) rim-enhancing collection within the right frontal lobe.

Based on sensitivities, the patient was discharged on an eight-week postoperative course of intravenous ceftriaxone 2 g twice daily and oral metronidazole 500 mg twice daily, followed by a high-dose oral amoxicillin 1 g every eight hours for a total of 12 months of antibiotic therapy. The patient had no sequelae or evidence of recurrence on imaging at the three-month (Figure 3) and eight-month follow-up (Figure 4).

## Discussion

We describe the case of a young, immunocompetent male who presented with intractable headaches and was found to have a cryptogenic brain abscess with multiple organisms, including *Actinomyces*, an organism that rarely affects the CNS. Cerebral infections are primarily caused by contiguous spread, often in the setting of cervicofacial surgeries or penetrative head trauma, or through hematogenous spread from cervicofacial infections [2]. However, this patient's workup was suggestive of neither etiology. The patient did not have bacteremia or signs of culture-negative endocarditis on the transesophageal echocardiogram to suggest hematogenous spread. The patient had no head, neck, or dental procedures or any evidence of odontogenic or sinus infection on imaging, suggesting a contiguous spread. While the patient did have a history of blunt head trauma, this was one year prior to symptom onset without evidence of fracture on imaging.

This patient did have an incidental finding of a PFO, which has been reported as a possible risk factor for cryptogenic brain abscesses in a few case reports. This is thought to be due to the bacteria bypassing the oxygen-rich pulmonary vascular bed and lymphatic system via a right-to-left shunt [5]. These reports frequently describe the PFO as a risk enhancer for brain abscesses, positing a mechanism through hematogenous spread in the setting of an extracranial infectious source or overall poor dentition leading to a transiently high bacterial load [5-7]. Current literature suggests that the probability of brain abscess formation in the setting of a PFO depends on the magnitude of the bacterial load as well as the size of the PFO. It is unclear if our patient's PFO is related to his CNS phenomena in the absence of other causative etiologies of infection.

## Conclusions

Our case presented a young, immunocompetent male with intractable headaches who was found to have a cryptogenic brain abscess with uncommon organisms on culture (HACEK spectrum and *Actinomyces*). Aside from a PFO, no evidence of endocarditis or predisposing cardiac or dental disease was found. Our case suggests that even in the absence of any clear sources of infection, cryptogenic brain abscesses can still occur sporadically in young, healthy patients. Future studies could assess the contribution of PFO as an antecedent risk factor for CNS abscess, specifically in patients with otherwise unknown etiology of illness.
